# 2837. Impact of Regulatory Guidelines on Therapeutic, Clinical, and Microbiological Success Rates in Uncomplicated Urinary Tract Infection: Results from a Two Phase 3 Randomized Controlled Trials of Oral Gepotidacin (EAGLE-2 and EAGLE-3)

**DOI:** 10.1093/ofid/ofad500.2447

**Published:** 2023-11-27

**Authors:** Florian Wagenlehner, Caroline R Perry, Thomas M Hooton, Nicole E Scangarella-Oman, Helen Millns, Emily Jarvis, Jeremy Dennison, Amanda Sheets, Salim Janmohamed

**Affiliations:** Justuf Liebeg University Diessen, Diessen, Hessen, Germany; GSK, Collegeville, PA, USA, Collegeville, Pennsylvania; University of Miami, Miami, Florida, USA, Coral Gables, FL; GlaxoSmithKline plc., Collegeville, Pennsylvania; GSK, Stevenage, UK, Stevenage, England, United Kingdom; GSK, Stevenage, UK, Stevenage, England, United Kingdom; GSK, Brentford, UK, Brentford, England, United Kingdom; GSK, Collegeville, PA, USA, Collegeville, Pennsylvania; GSK, Brentford, UK, Brentford, England, United Kingdom

## Abstract

**Background:**

For noninferiority RCTs in uUTIs, the latest US Food and Drug Administration (FDA; 2019)/European Medicines Agency (EMA; 2018) guidance recommends a primary endpoint of therapeutic response (combined clinical and microbiologic response) with success defined by full symptom resolution plus microbiological eradication from ≥ 10^5^ to < 10^3^ CFU/mL. Historically, less stringent criteria (clinical or microbiological [not combined], allowing symptom improvement/resolution) and various endpoints were used; success rates of 70–92% have been reported for NTF (**Table 1**). Gepotidacin, a novel, first-in-class, triazaacenaphthylene, bactericidal ABX has been investigated for uUTI treatment in 2 RCTs, EAGLE-2 (E-2; NCT04020341) and -3 (E-3; NCT04187144) following FDA/EMA guidance. Here, we demonstrate the impact of new endpoint guidance on the RCT results.

**Figure:**
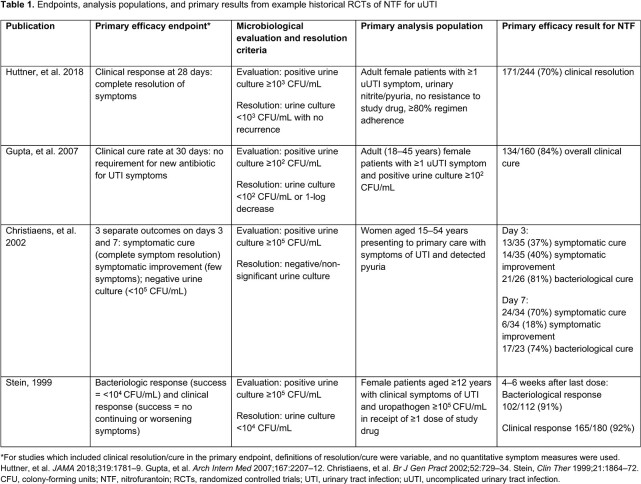

**Methods:**

E-2 and -3 were global Phase 3, parallel-group, double-blind, noninferiority (10% margin) RCTs comparing the efficacy and safety of oral gepotidacin (1500mg BID, 5 days) vs NTF (100mg BID, 5 days) in female patients (≥ 12 years) with uUTI. Using the complete study dataset, we assessed all patients who received ≥ 1 dose of study drug and had 1–2 qualifying uropathogens (≥ 10^5^ CFU/mL) susceptible to NTF. TOC was 10–13 days post first dose, FU was 28 (±3) days.

**Results:**

In E-2/-3, the primary endpoint was assessed in 634/567 patients (41/35% of ITT; **Table 2**). At TOC in E-2/-3, clinical success was 66.7/68.2% for gepotidacin, and 65.8/63.6% for NTF. Microbiological success in E-2/-3 was 72.6/72.9% for gepotidacin, and 66.8/57.5% for NTF. TOC therapeutic success rates (E-2/E-3) were lower than clinical or microbiological rates for both treatments: 51.8/58.9% for gepotidacin, 47.0/44.0% NTF (**Table 3**). Exploratory analysis replacing clinical ‘resolution’ with ‘near success’ increased TOC therapeutic success rates by 6.2–9.5% (**Table 3**). Use of non-study ABX for uUTI was substantially lower than the overall rate of therapeutic failure at TOC and FU.

**Figure:**
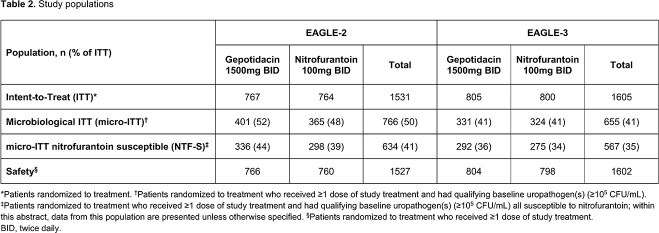

**Figure:**
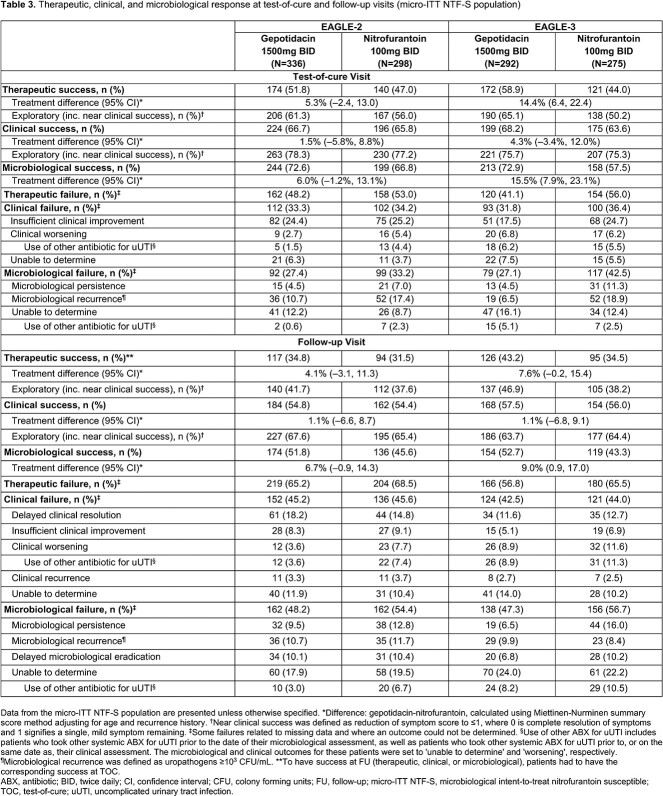

**Conclusion:**

In these 2 RCTs using recent regulatory criteria that set a high threshold for uUTI treatment efficacy, therapeutic and clinical success rates were lower than some historic studies. Contemporary study results using new guidance cannot be directly compared with historical studies.

**Disclosures:**

**Florian Wagenlehner, MD**, Achaogen: Advisory Board member, study participation|Astellas: Honoraria|AstraZeneca: Honoraria|AstraZeneca: Advisory Board member|Biomedical Advanced Research and Development Authority (BARDA): Grant/Research Support|Bionorica: Honoraria|Bionorica: Meeting/travel support, study participation|Deutsches Zentrum für Infektionsforschung (DZIF): Study participation|Enteris BioPharma: Study participation|Everest Medicines: Grant/Research Support|German S3 guideline Urinary tract infections: Board Member|Glaxo Smith Kline: Advisor/Consultant|Glaxo Smith Kline: Honoraria|Glaxo Smith Kline: Consulting fees, meeting/travel support, advisory board member, principal investigator in a GSK-sponsored study|Global Antibiotic Research and Development Partnership (GARDP Foundation): Grant/Research Support|Guidelines European Association of Urology: Infections in Urology: Board Member|Helperby Therapeutics: Study participation|Janssen: Honoraria|Janssen: Advisory Board member|Klosterfrau: Honoraria|LeoPharma: Advisory Board member|MerLion: Advisory Board member|MIP Pharma: Honoraria|MSD: Advisory Board member|OM Pharma/Vifor Pharma: Advisory Board member, study participation|OM-Pharma: Honoraria|Pfizer: Honoraria|Pfizer: Advisory Board member|RosenPharma: Advisory Board member|Shionogi: Advisory Board member, study participation|Speaker research group German research foundation (DFG) Bacterial Renal Infections and Defense (FOR 5427): Study participation|Spero Therapeutics: Advisor/Consultant|Spero Therapeutics: Consulting fees|University Hospital Giessen and Marburg GmbH, and Justus Liebig University, Germany: Employee|Venatorx Pharmaceuticals, Inc.: Advisor/Consultant|Venatorx Pharmaceuticals, Inc.: Grant/Research Support|Venatorx Pharmaceuticals, Inc.: Consulting fees, Advisory Board member **Caroline R. Perry, PhD**, GSK: Employee and shareholder **Thomas M. Hooton, MD**, GSK: Advisor/Consultant **Nicole E. Scangarella-Oman, MS**, GSK: Employee and shareholder **Helen Millns, PhD**, GSK: Employee and shareholder **Emily Jarvis, MSc**, GSK: Employee and shareholder **Jeremy Dennison, MD**, GSK: Employee and shareholder **Amanda Sheets, PhD**, GSK: Employee and shareholder **Salim Janmohamed, MD**, GSK: Employee and shareholder

